# Multidisciplinarity and Transdisciplinarity as Current Trends in Otorhinolaryngology and Head and Neck Pathology

**DOI:** 10.3390/medicina58111661

**Published:** 2022-11-16

**Authors:** Daniela Vrinceanu, Codrut Sarafoleanu, Mahmut Tayyar Kalcioglu, Adriana Neagos

**Affiliations:** 1 ENT Department, Carol Davila University of Medicine and Pharmacy, 020021 Bucharest, Romania; 2 Department of ORL & HNS, Istanbul Medeniyet University, 34720 Istanbul, Turkey; 3 ENT Department, George Emil Palade University of Medicine, Pharmacy, Science and Technology of Targu Mures, 540142 Targu Mures, Romania

The specialty of otorhinolaryngology and cervicofacial surgery has experienced accelerated development in recent decades through the development of the techniques and technologies involved. Thus, from the classic image of the ENT specialist who operated on tonsils and adenoids during in-office procedures in the late 1970s, we have reached a type of ENT specialist and surgeon who tackles difficult and complex fields and pathologies, continuously exceeding the predefined limits of the specialty. A significant challenge for otolaryngologists was the COVID-19 pandemic and the infection with the SARS-CoV-2 virus; in this context, our specialty is at the entrance gate of the virus, generating new clinical pictures but also developmental directions for topical nasal vaccines, assuming and continuing collaboration with infectious disease specialists [1]. Endoscopic rhinosinusal surgery and neuro-navigation systems have progressively pushed the limits of endonasal surgery, especially for sinonasal tumors and transnasal surgery, which involves the skull base approach and working with a neurosurgeon [2]. Obstructive sleep apnea syndrome (OSA) involves teams with a pulmonologist, maxillofacial surgeon, and ENT surgeon, with results that lead to an increase in the quality of life of our patients [3].

Associated with classically approached head and neck pathology, several chapters of pathology are considered ”no man’s land” and addressed by several surgical specialties. An example is the infratemporal fossa approach, frequently shared between the neurosurgeon, the maxillofacial surgeon, and the ENT surgeon. Another example is cervicofacial vascular malformations, a delicate chapter often burdened with disappointments and limitations shared between the vascular surgeon, maxillofacial surgeon, ENT surgeon, and plastic surgeon. Craniofacial and cervical trauma involves, due to the loco-regional anatomical and functional adjacencies, a multidisciplinary approach because it involves an ENT surgeon, maxillofacial surgeon, ophthalmologist, neurosurgeon, plastic surgeon, vascular surgeon, thoracic surgeon, and anesthesiologist. Thyroid surgery has to be completed together with an endocrinologist and sometimes with a thoracic surgeon [4].

Of course, the most complex field that constantly transcends boundaries is represented by head and neck oncology. Head and neck oncological surgery with tumor ablative time and reconstructive time to limit aesthetic and functional deficits requires joint-trained teams involving all types of surgeons already mentioned, along with an anesthetist, imager, and pathologist [5]. Adjuvant or neoadjuvant therapy involves tumor boarding with an oncologist, radiotherapist, speech therapist, and psychologist, and the list is permanently open [6].

In solving complex cases, a single surgical specialty is seldom enough. It is necessary to develop a multidisciplinary perspective, which involves bringing together several skills in diagnosing and treating a patient. However, multidisciplinarity goes beyond gathering consultations from several specialties in a given case, an undoubtedly important aspect from a medico-legal point of view, but also finding an optimal therapeutic solution. Modern pathology requires transdisciplinary case-solving, which represents the integration of skills introduced by mixed teams of different specialists that address head and neck pathology without going beyond the legal limits of medical practice that define malpraxis [7]. The multidisciplinary teams acquire training over time, according to the solution of the cases, learning from each other from successes and cases with a less favorable evolution. In essence, multidisciplinarity also includes the ability to see the patient through the eyes of a colleague from another specialty; to intraoperative mobility with successive operating times; to sequenced treatment stages with the essential aim of identifying the optimal therapeutic solution, often individualized for each patient, because certainly many minds think better than one.

We proposed that the multidisciplinary and transdisciplinary approach to head and neck pathology be the theme of this Special Issue entitled “Current trends in Otorhinolaryngology and Head and Neck pathology”. We invite specialists in this pathology to cross the borders between specialties, submit research articles to share clinical and surgical experience, generate discussions that lead to progress, and enrich our daily practice.

## Data Availability

Not applicable.
